# Supplementary figures and images for: Transcriptome analysis of Catarina scallop (Argopecten ventricosus) juveniles treated with highly-diluted immunomodulatory compounds reveals activation of non-self-recognition system
Source: PLoS One. 2020 May 14;15(5):e0233064. doi: 10.1371/journal.pone.0233064 (PMC7224555; doi:10.1371/journal.pone.0233064)

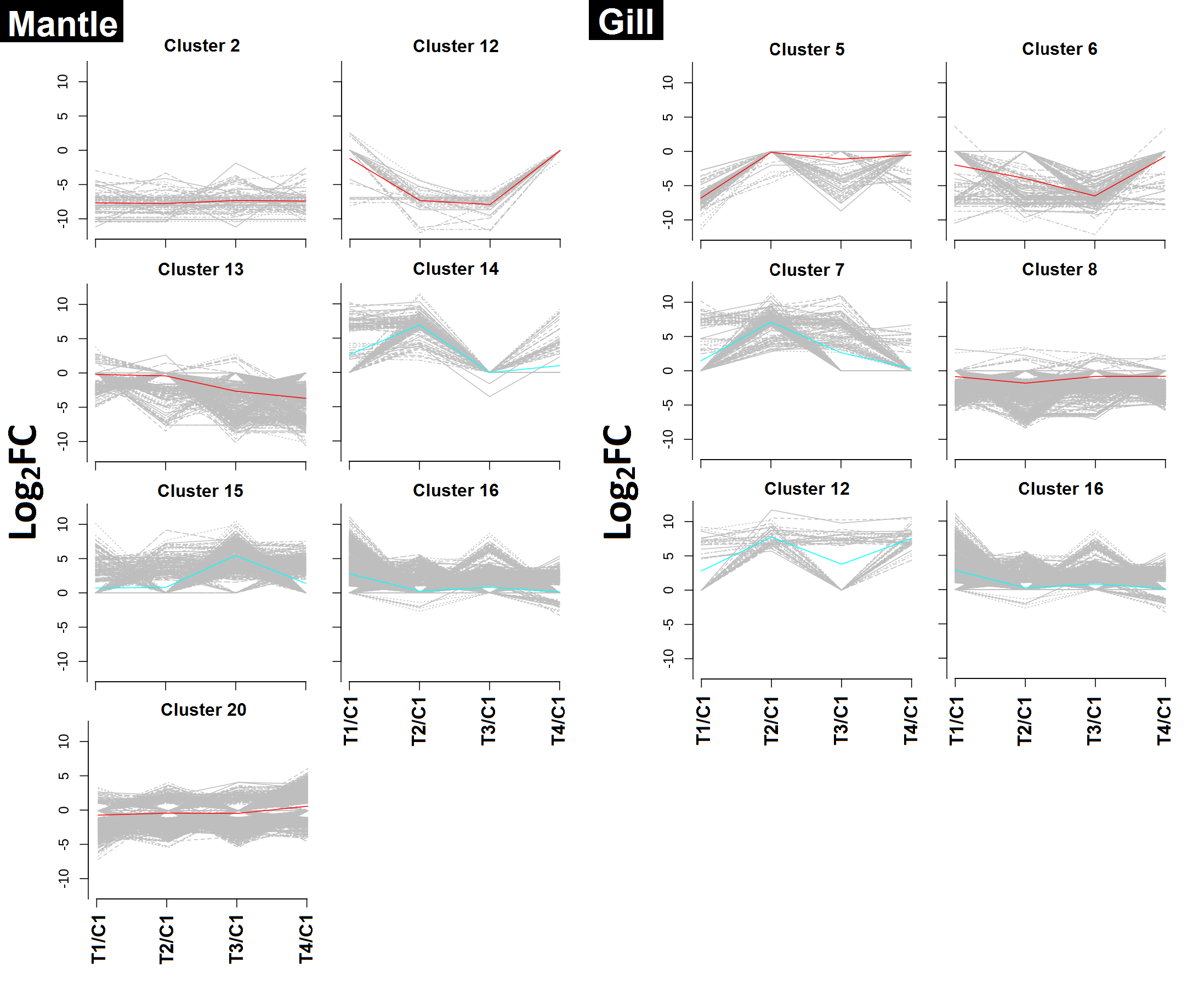

Supplement: S1 Fig — Biological process or metabolic pathways with more than five transcripts are shown in graph with grey lines with the average datum behaviour represented by a red line. (TIF) [file pone.0233064.s001.tif]

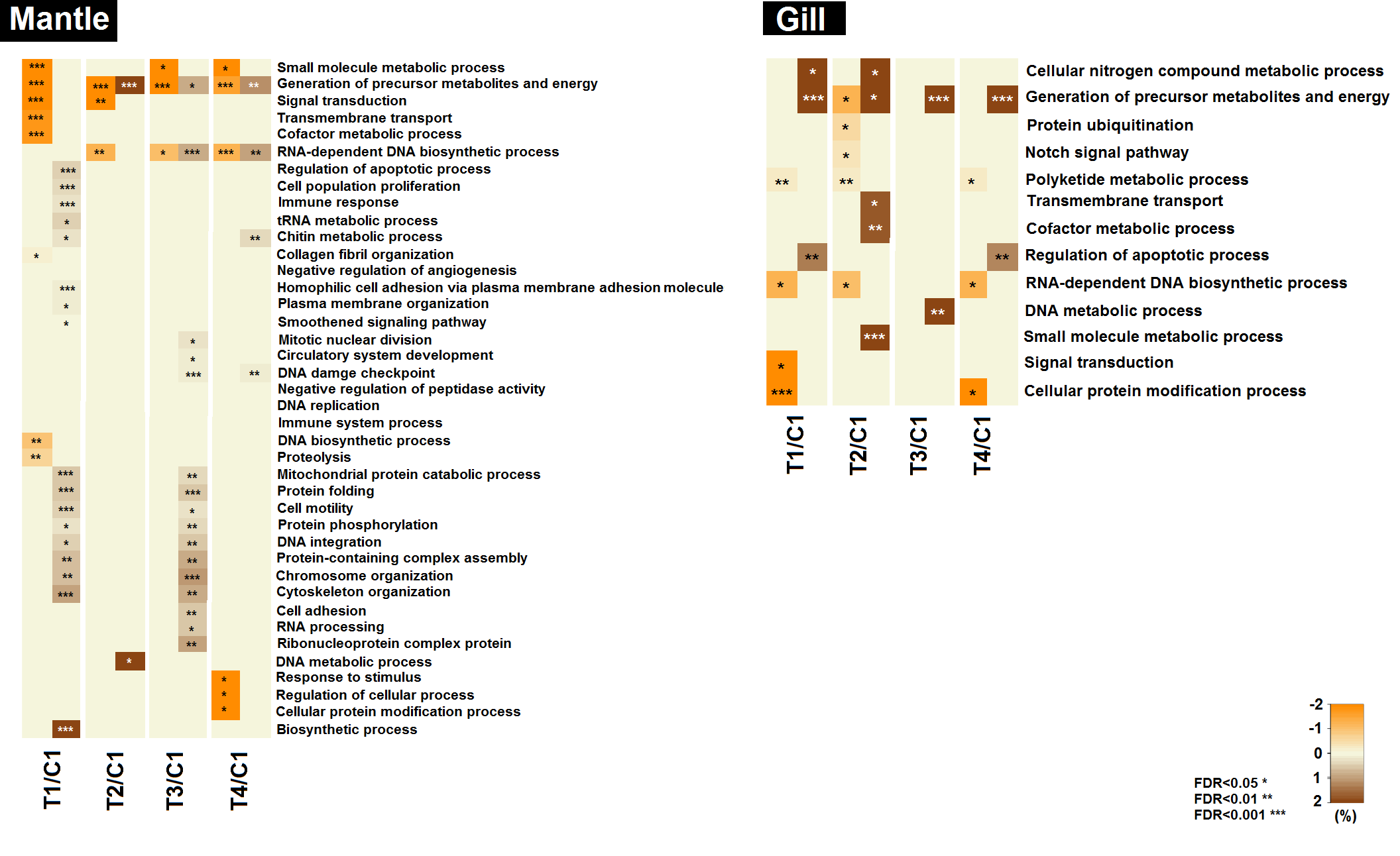

Supplement: S2 Fig — The colour key indicates from beige to brown the increasing percentages of genes representing each up-regulated category and from beige to orange the increasing percentages of genes representing each down-regulated category. Blast2go specific filter was applied to the enrichment analysis to eliminate general categories. Contrasting conditions referred to all treatments evaluated vs control. (TIF) [file pone.0233064.s002.tif]

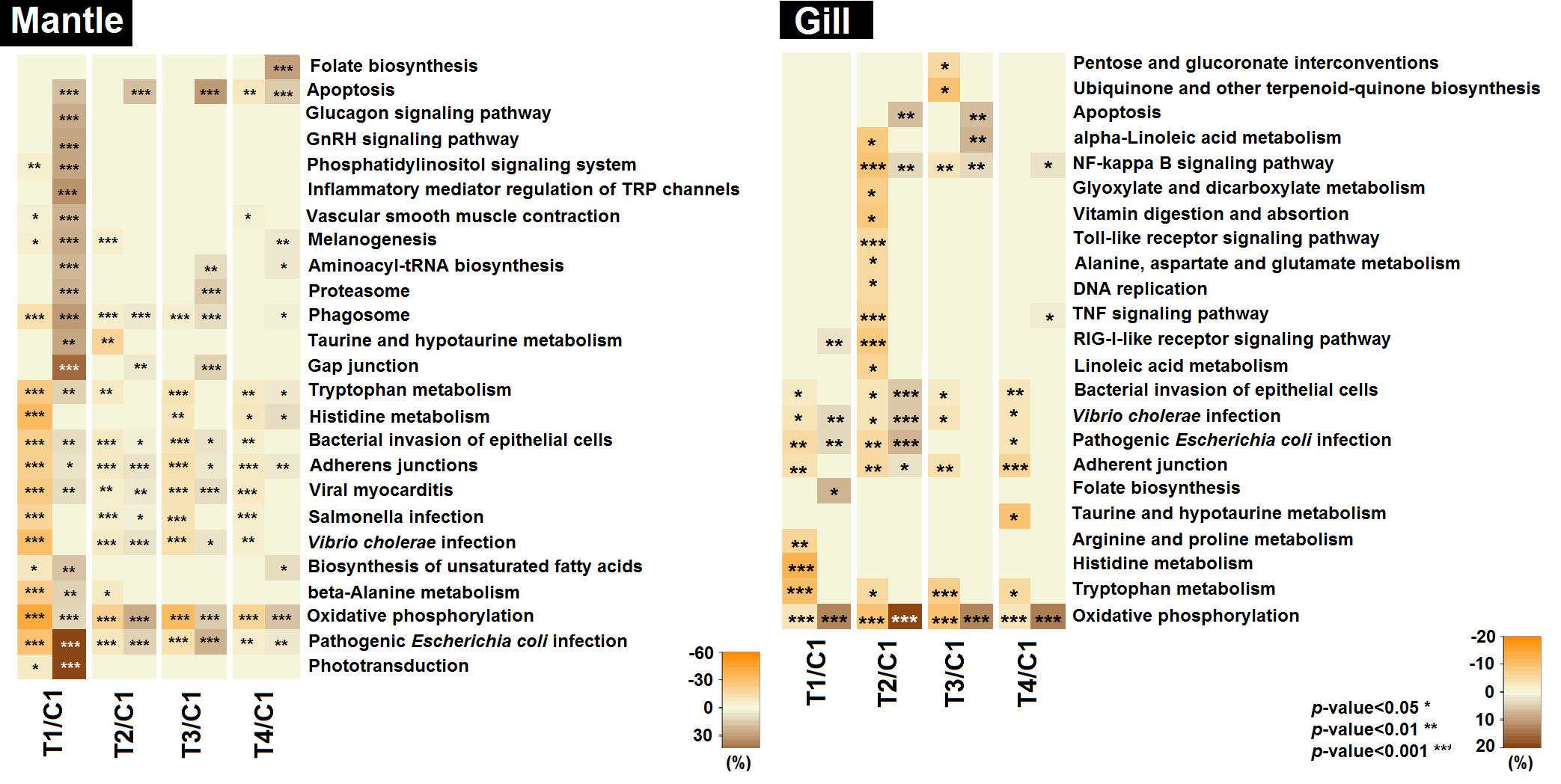

Supplement: S3 Fig — The colour key indicates from beige to brown the increasing percentages of genes representing each up-regulated category and from beige to orange the increasing percentages of genes representing each down-regulated category. Blast2go specific filter was applied to the enrichment analysis to eliminate general categories. Contrasting conditions referred to all treatments evaluated vs control. (TIF) [file pone.0233064.s003.tif]
