# Supplementary material for: Transcriptome analysis of Catarina scallop (Argopecten ventricosus) juveniles treated with highly-diluted immunomodulatory compounds reveals activation of non-self-recognition system
Source: PLoS One. 2020 May 14;15(5):e0233064. doi: 10.1371/journal.pone.0233064 (PMC7224555; doi:10.1371/journal.pone.0233064)
Supplement: Attachment — Submitted filename: Resived PONE-D-20-00449 Lopez-Carvallo et al.pdf [file pone.0233064.s008.pdf]

Transcriptome analysis of Catarina scallop (*Argopecten ventricosus*) juveniles treated with highly-diluted immunomodulatory compounds reveals activation of non-self-recognition system

Scallop transcriptome and immunomodulatory compounds

Jesús Antonio López-Carvallo[1], José Manuel Mazón-Suástegui[1], Rosa María Morelos-Castro[2], Dariel Tovar-Ramírez[3], Fernando Abasolo-Pacheco[4], Miguel Ángel Hernández-Oñate[5,*], Guadalupe Fabiola Arcos-Ortega[2,*]

[1]Laboratorio Experimental de Cultivo de Moluscos, Centro de Investigaciones Biológicas del Noroeste, La Paz, B.C.S., México.

[2]Laboratorio de Imunogenómica Marina, Centro de Investigaciones Biológicas del Noroeste, La Paz, B.C.S., México.

[3]Laboratorio de Fisiología Comparada y Genómica Funcional, Centro de Investigaciones Biológicas del Noroeste, La Paz, B.C.S., México.

[4]Facultad de Ciencias Agrarias, Universidad Técnica Estatal de Quevedo,

Quevedo, Los Ríos, Ecuador.

[5]*Programa de Cátedras CONACYT, Centro de Investigación en Alimentación y Desarrollo A.C, Hermosillo, Sonora, México.*

*CORRESPONDING AUTHORS:
E-mail: farcos04@cibnor.mx (GFAO)
E-mail: miguel.hernandez@ciad.mx (MAHO)

Abstract

Marine bivalve hatchery productivity is continuously challenged by apparition and propagation of new diseases, mainly those related to vibriosis. Disinfectants and antibiotics are frequently overused to prevent pathogen presence, generating a potential negative impact on the environment. Recently, the use of highly diluted compounds with immunostimulant properties in marine organisms has been trailed successfully to activate the self-protection mechanisms of marine bivalves. Despite their potential as immunostimulants, little is known about their way of action. To understand their effect, a comparative transcriptomic analysis was performed with *Argopecten ventricosus* juveniles. The experimental design consisted of four treatments formulated from pathogenic *Vibrio* lysates at two dilutions: [(T1) *Vibrio parahaemolyticus* and *Vibrio alginolyticus* 1D; (T2) *V. parahaemolyticus* and *V. alginolyticus* 7C]; minerals [(T3) PhA+SiT 7C], scorpion venom [(T4) ViT 31C]; and one control (C1) hydro-alcoholic solution (ethanol 1%). The RNA sequencing (RNAseq) analysis showed a higher modulation of differentially expressed genes (DEG) in mantle tissue compared to gill tissue. The scallops that showed a higher number of DEG related to immune response in mantle tissue corresponded to T1 (*V. parahaemolyticus* and *V. alginolyticus* lysate) and T3 (S*ilicea terra* - Phosphoric acid). The transcriptome analysis allowed understanding some interactions between *A. ventricosus* juveniles and highly-diluted treatments.

KEYWORDS: RNAseq, Silica, Phosphoric acid, Immune response, *Argopecten ventricosus*, *Vibrio* lysate.

Introduction

Marine bivalve hatchery production has been challenged by apparition and propagation of new diseases [1], mainly those related with *Vibrio spp.* [2,3], leading to high mortality and economical losses. Recent studies have suggested that proliferation of pathogenic vibrio species will increase in a near future due to sea surface water warming [4,5], and most of the time, hatchery conditions have been favourable to *Vibrios spp.* [6]. Traditionally, hatcheries around the world have routinely implemented the prophylactic application of antibiotics and disinfectants to prevent proliferation or kill pathogenic bacteria, as standard prophylactic or therapeutic procedures [7,8].

Chemotherapeutic applications have been restricted by governmental institutions [2] for their undesirable effects, such as selection of antibiotic-resistant bacteria [7,8], their implications in dissolution of mercury compounds when contaminated water is in contact with marine sediment [9] and alterations in the beneficial gastrointestinal microbiota of cultured organisms [10].

Immunostimulants have been proposed as promising and sustainable alternatives to avoid massive bivalve mortalities in hatchery and reduce or

replace the use of antibiotics [11]. Some immunomodulatory compounds can prophylactically activate self-protection of the organisms instead of killing the pathogen by chemical compounds, which may compromise health of the organisms and environment. Within the immunostimulant category, highly-diluted immunomodulatory compounds (HDIC) formulated from bacterial lysates, scorpion venom, silica and Phosphoric acid have been recently suggested as eco-friendly and sustainable options to activate the immune system without affecting the general condition index of marine organisms [12]. HDIC are inexpensive to produce because serial dilutions at decimal (D, 1:10) or centesimal (C, 1:100) scale [13] are used, and the action mode of these treatments depends on their dilution grade. Low decimal dilution (D) effects are attributed to diluted molecules and nanoparticles while high centesimal dilution (C) effects are attributed to nanoparticles and electromagnetic fields [14], which are sensed by traditional molecular receptors or photoelectrochemical sensing system activated by ultra-weak photo emission signals [15]. These highly-diluted molecules work as low intensity danger signals that generate endogenous amplification by hormesis effect, time-dependent sensitisation and stochastic resonance process [16,17].

Low concentrations of attenuated bacteria [18,19] have already proven to activate immune response in different organisms and highly-diluted concentrations of bacterial lysates, *Silicea terra*, Phosphoric acid and Vidatox® (Habana, Cuba) have been successfully used in marine organisms to improve

response against pathogens [12]. Previous studies have demonstrated that survival of *Argopecten ventricosus* juveniles increased when organisms were previously therapeutically treated by *Silicea terra* and Phosphoric acid 31C, and then challenged against highly pathogenic bacteria, such as *Vibrio parahaemolyticus* [20]. Furthermore, increase in growth, enzymatic antioxidant activity, and haemocyte proliferation were reported when juvenile scallops were treated prophylactically with diluted and highly-diluted pathogenic bacterial lysates (1D and 7C) for a 21-day period [21]. Although those immunomodulatory compounds are known to allow organisms to activate immune response and increase survival when they face pathogen infections, little is known about how they work on the immune response of marine organisms.

Transcriptomic (RNA-seq) analysis allows discovering potential and novel action mechanisms. Particularly, these analyses have provided information to analyse complex relations among disease, drugs, and organisms [22]. Moreover, RNA-seq has been used in marine bivalves to understand physiological stress [23,24], toxicological effects [25,26], and resistance to disease and immunology [27,28]. In this sense, the RNA-seq analysis helps to understand the molecular response activated by the effect of time-dependent diluted immunomodulatory treatments, which allowed identifying the down- and up-regulated genes of juvenile scallops in response to HDIC.

Because the immune defence mechanisms in response to HDIC formulated by bacterial lysates, *Silicea terra*, Phosphoric acid and Vidatox® (Habana, Cuba) remain unknown, this study performed a comparative analysis of the mantle and gill transcriptome profile treated with those HDIC. These data will provide important information about the genes and mechanisms that are being regulated in *A. ventricosus* scallop and contribute to understanding how HDIC acts on marine organism response.

This study also provided for the first time whole transcriptome data of the Catarina scallop *A. ventricosus* juveniles, which was selected as a model organism because of its importance as fishing resource in Baja California Sur, Mexico. Its wild populations have been decreasing throughout the years [29], and spat production at hatchery level faces high mortality events as it is a species highly susceptible to *vibrosis* [30], one of the main bacteria present in hatchery culture water.

Materials and Methods

Scallop acquisition and experimental design

The non-governmental association Noroeste Sustentable (NOS) provided 1500 juvenile scallops (average length 1.98 ± 0 .1 cm) from Bahia de La Paz, Mexico to perform the experiment. Scallops were acclimated for one week in a nursery upwelling recirculating system with constant food

concentration of 150 000 cell mL^{-1} (*Isochrysis galbana; Chaetoceros calcitrans*; cellular proportion 1:1). Filtered seawater (1 μm, activated carbon and ultraviolet (UV) irradiation) at 24°C and 38.5 ± 0.5 UPS salinity were continuously supplied to allow water change totally every day.

After acclimatization, scallops were transferred to 15 experimental units (36-L container) with 52 scallops each (three experimental units per treatment). During the experimental period (21 days), scallops were kept in an open-recirculating flow system that provided filtered and treated seawater (1 μm, activated carbon and UV irradiation) with a blend of *I. galbana* and *C. calcitrans* microalgae (199 607 794 cell/organism/day) at 23.5 ± 0.5°C, and 38.5 ± 0.5 UPS salinity. A 21-day experimental period was sufficient to strengthen the immune system of this species with HDIC-based treatments [20].

Four experimental HDIC treatments [T1: *V. parahaemolyticus* and *V. alginolyticus* lysate diluted at 1:10 (1D); T2: *V. parahaemolyticus* and *V. alginolyticus* lysate diluted at 1: 10^{-14} (7C); T3: *Silicea terra* (Similia®, Farmacia Homeopática Nacional®, CDMX, MX) and Phosphoric acid (Similia®, Farmacia Homeopática Nacional®, CDMX, MX) diluted at $1X10^{-14}$ (7C); T4: Vidatox® (Habana, Cuba) diluted at $1X10^{-62}$ (31C)], and one control treatment [C1: hydro-alcoholic solution (1%)] were assayed by triplicate under laboratory conditions. All HDIC or control treatments were supplied to experimental units as used in our previous studies [20,21]. The open water and food flow was cut

three hours a day to favour treatment uptake through the mantle and gill tissues. All treatments were added in liquid form directly to seawater in each experimental unit (100 μl L^{-1}). Treatments T1, T2 and T3, which consisted of two HDIC each, were provided alternately.

At the end of the experiment, organisms for each experimental condition were taken. The mantle and gill tissues of three sampled scallops of each experimental replicate unit were excised, separately fixed in RNAlater solution (#AM7020, ThermoFisher, Scientific, Waltham, MA, USA) and stored at -80°C for ~~posterior~~ analysis. Mantle and gill tissue were selected as target organs because they have been highly implicated with immune response and have been related with pattern recognition receptors in marine bivalves [31,32].

Highly-diluted immunomodulatory compounds (HDIC) formulation

All treatments and control were made following the methodology previously reported for HDIC preparation [21,33], which is described below. T1 (ViP 1D + ViA 1D) and T2 (ViP 7C + ViA 7C) were developed at Centro de Investigaciones Biológicas del Noroeste (CIBNOR), La Paz, Baja California Sur, Mexico and formulated in decimal (T1) or centesimal (T2) dilution/succussion (agitated) from a concentrate of pathogenic strains of *V.*

parahaemolyticus (CAIM 170; www.ciad.mx/caim » ViP) and *V. alginolyticus* (CAIM 57; www.ciad.mx/caim » ViA). These strains were isolated from high mortality events in marine bivalves. For each strain, a concentrated solution was prepared from wet biomass obtained by centrifugation (13 000 rpm, 4°C, 20 min) of 2 L of *Vibrio* culture in marine broth 2216 (105 × 10^{-6} CFU mL-1). Wet biomass of each strain (15 mL) was fully inactivated by three freeze-unfreeze cycles of −80 and 24°C; strain biomass was vortexed at 3200 rpm (Benchmark mixer™, Benchmark Scientific Inc. Sayreville, NJ, USA) between each cycle for ~~two~~ 2 min. The inactivated product was topped up to the original culture volume (2 L) using ethanol 87° (Similia® purchased at Farmacia Homeopática Nacional®, CDMX, MX) and vortexed at 3200 rpm (Benchmark mixer™, Benchmark Scientific Inc. Sayreville, NJ, USA) for ~~two~~ 2 min to get a final concentrated solution, from which decimal (D, 1:10) and centesimal (C, 1:100) dilutions were prepared by serial successive dilution/succussion process (3200 rpm, ~~two~~ 2 min, Benchmark mixer™, Benchmark Scientific Inc. Sayreville, NJ, USA) until working solution 1D (1X10) and 7C ($1X10^{14}$) had been reached.

Treatments T3 (PhA 7C + SiT 7C) and T4 (ViD 31C) were made from commercial drugs diluted in ethanol 87°. T3 consisted of centesimal (C) preparations (1:100 dilution/succussion) of the commercial drugs *Phosphoricum acid*® 6C (Similia®, CDMX, MX; dilution 1 × 10^{12}) and *Silicea terra*® 6C (Similia®, CDMX, MX; dilution 1 × 10^{12}) while T4 (ViD 31C)

Commented [MH1]: Need reference

Commented [MH2]: Which company, country

Commented [MH3]: Did you make sterility test for the HDIC solutions originated from the used vibrios??

consisted of a centesimal dilution (1:100 dilution/succussion) of the commercial drug Vidatox® (Habana, Cuba) 30C (Labiofam®, Habana, Cuba; dilution 1×10^{60}) made from blue scorpion *Rhopalurus junceus* venom. To prevent potential ethanol side effects, distilled water was used as a final dilution vehicle for all HDIC working solutions.

RNA extraction and cDNA synthesis

To extract RNA, the tissue (mantle and gill) samples from three scallops (~100 mg) were collected and pooled. Each pool was considered as the biological sample. For the Real-Time quantitative polymerase chain reaction (RT-qPCR) analysis, three pools of each tissue were used per treatment (Fig 1A). For transcriptomic analysis, two pools (~100 mg each) were used per treatment (Fig 1A). The RNA extraction was assessed following the methodology previously described for the scallop *Nodipecten subnodosus* RNA extraction [34], using the TriPure reagent (Roche Diagnostics, Indianapolis, IN, USA), followed by ethanol/chloroform purification and DNAse cleaning. Samples were determined for RNA concentration using a NanoDrop 2000/2000c® spectrophotometer (Thermo Fisher Scientific, Waltham, MA, USA), and electrophoresis was performed to evaluate RNA quality. Good quality RNA from a total of 20 Pools (two per treatment per tissue) were sent to the Laboratorio Nacional de Genómica para la Biodiversidad (LANGEBIO,

Laboratory of Genomics Services, CINVESTAV, Campus Irapuato, GTO, MX) to create and sequence the RNA-seq libraries using the Illumina Next-Seq® (San Diego, CA, USA). For RT-qPCR analysis, the cDNA synthesis was performed following the methodology previously reported to quantify gene relative expression levels in *N. subnodosus* [34].

Fig 1. Sampling collection and data analysis strategy. (A) Work diagram for sample pooling. Each diagram represents the pooling strategy for each experimental condition. Purple and yellow colours denote organisms used for transcriptome pools (three organisms per replicate) and blue denotes organisms used for qPCR pools (three organisms per replicate); (B) RNAseq analysis strategy. For each tissue, treatments were separately compared against the control. T1: *V. parahaemolyticus* and *V. alginolyticus* lysate 1D; T2: *V. parahaemolyticus* and *V. alginolyticus* lysate 7C; T3: *Silicea terra* and Phosphoric acid 7C; T4: Vidatox® (Habana, Cuba) 31C, and control treatment C1: hydro-alcoholic solution (1%).

Illumina sequencing

For RNA-seq library preparation, the two RNA pools were selected from mantle and gill of each experimental condition. A total of 20 libraries were prepared using the RNA extractions. In addition, 12 libraries were prepared

with the organisms treated with HDIC formulated by sodium metasilicate - Phosphoric acid 1D plus two controls (C2: diluted ethanol 1:100 and C3: nothing added) to increase assembly and annotation quality. Libraries were validated by 2100 Bioanalyzer Instrument (Agilent, Santa Clara, CA, USA) and sequenced with Illumina NextSeq® (San Diego, CA, USA) platform on mode paired-end (2 X 150 high). Generation of libraries and sequencing were performed by LANGEBIO (Laboratory of genomics services, CINVESTAV, Campus Irapuato, GTO, MX).

Transcriptome analysis and annotation

Commented [MH4]: This title of this section may be changed to "**Transcriptome, annotation and statistical analyses"**

The raw reads obtained by the Illumina NextSeq® platform (San Diego, CA, USA) were first analysed with the FastQC software [35] to determine quality sequences; then, the reads were filtered using the Trimmomatic program [36] by removing low-quality reads (Q < 25), ambiguity ("N"), reads under 50 bp and trimming the adapters. The high-quality filtered reads were used to perform the *de novo* transcriptome assembly with Trinity software [37] with the "non-normalized reads" option. This process was performed in a WorkStation with 10 cores and 512 GB RAM. Once the assembly process was completed, the quality and integrity of the transcriptome were analysed. The high-quality filtered reads were mapped to assembled transcripts with RSEM software which used Bowtie2 for the alignment [38], and the expression values

were normalised to transcripts per million (TPM) for each transcript. Those with low expression values (TPM < 0.01) were removed. The assembled transcripts were compared against the non-redundant nucleotide and protein databases of the National Center for Biotechnology Information (NCBI) using BLAST with an e-value < 1e^{-5}, sequences with hit to viruses, bacteria, fungus, and so on (considered contaminant sequences) were removed using the SeqClean tool [39]. Transcriptome redundancy was first removed by CD-Hit using 97% of identity as clustering threshold [40] and then by iAssembler program [41]. The statistic, quality and completeness analyses of the assembled transcriptome were performed using the PRINSEQ [42], TransRate [43], and BUSCO [44] tools.

To determine the functional annotation based on homology, the filtered transcriptome was compared against the public protein non-redundant nucleotides (NCBI), Swiss-Prot and refSeq databases using BLASTX (e-value < 1e^{-5}). In addition, a BLASTX (e-value < 1e-5) against predicted proteins of the *Crassosrea gigas*, *Crassostrea virginica*, *Mytilus galloprovincialis* and *Mizuhopecten yessoensis* genomes were performed. The functional annotation was assigned according to the BLAST hits. Finally, the functional categorisation was designated based on the comparison with the Gene Ontology (GO), InterPro and Kyoto Encyclopaedia of Genes (KEGG) databases using the BLAST2GO software [45]. The raw reads were deposited in the Gene Expression Omnibus of NCBI under accession number

PRJNA596225.

Differential expression analysis and enrichment analysis

Only were 20 libraries corresponding to the treatments (T1, T2, T3, T4) and control (C1) considered for the posterior analysies. High-quality alignment of the filtered reads to the filtered transcriptome and quantification of reads were carried out using the RSEM software [38]. Raw counts of each transcript of each library were used to generate a matrix with all experimental conditions and replicates. These raw counts were normalised to fragments per kilobase per million mapped reads (FPKM). To determine dispersion between samples and biological replicates, the Pearson correlation value was calculated between all experimental libraries. Only were libraries with Pearson's correlation > 0.88 between replicates kept for subsequent analyses.

Once every biological replicate was validated, transcripts lowly expressed were removed and only transcripts with a FPKM > 0.3 in at least two replicates of at least one treatment were kept, such as edgeR that reduced analysis quality when very low expressed genes were used [46]. Differentially expressed genes in Catarina scallop juveniles treated with the HDIC (Fig 1B) versus control without treatment were identified with the edgeR package [46], using a statistical method based on the generalised linear model (GLM). To

estimate the variance between samples, tagwise dispersion was calculated. Transcripts with FDR < 0.01 and Log_2FoldChange >1 (Log_2FC) were considered differentially expressed. DEG transcripts were grouped by tissue as down- and up-regulated for each treatment, and then Venn diagrams were generated including all the comparisons between DEG of treatments.

Gene Ontology and KEGG categories enrichment analyses were performed using BLAST2GO [45] and KOBAS [47], respectively, with FDR < 0.05. The function to most specific terms was used to reduce the result-set of over-represented GO terms. Heatmaps and hierarchical clustering were carried out using the gplots and hclust in R [48].

RT-qPCR Validation

To validate differential expression from RNAseq data, 10 target genes (Log_2FC > 1.5 and FDR < 0.01) and four constitutive genes (CV% < 0.3, Log_2FC between -1.5 and +1.5 and FPKM > 0.3) from the transcriptome were selected (S1 Table). Primers were designed using the software Primer3 [49] and then analysed with OligoAnalyzer Tool [50]. The most stable of the constitutive genes for each tissue was selected using RefFinder software [51] to express relative expression. Primers were evaluated and target sequences validated by sequencing analysis (Macrogen, Seoul, Korea).

For the RT-qPCR analysis, three biological replicates were assessed

per treatment (each biological replicate was performed by triplicate) using previously synthesised cDNA from mantle and gill. RT-qPCR analysis was performed as previously reported to quantify gene relative expression levels in *N. subnodosus* [34] following Minimum Information for Publication of Quantitative Real-Time PCR Experiments (MIQE) guidelines [52]. Percentage of efficiency of each primer pair was obtained from efficiency curves from six serial dilutions (1:5) [52]. Reactions of RT-qPCR were carried out with the equipment using 2X EvaGreen® (ThermoFisher, Scientific, Waltham, MA, USA) PCR mix. Reaction mixtures were made in a 15 µL, including 10 µL mix (0.45 U of GoTaq Flexi DNA polymerase (Promega, Madison, WI, USA), 2.5 mM MgCl2, 1x Go Taq Flexi Buffer, 0.2 mM dNTP Mix (Promega, Madison, WI, USA), 2x EvaGreen fluorescent dye (Biotium, Inc. Fremont, CA, USA), 0.15–0.45 µM each primer) and 5µL cDNA (diluted to 50 ng μL^{-1}). The amplification parameters were 4 min at 94°C, followed by 39 cycles of 9 s at 95°C, 30 s at 60°C and 30 s at 72°C; finally, a melt curve was added to confirm amplification of specific products. Data were analysed using Ct values and the $2^{-\Delta\Delta Ct}$ method [53] and relative expression was normalised to the abundance of sodium/potassium-transporting ATPase subunit alpha-like gene for mantle tissue and cAMP-dependent protein kinase catalytic subunit-like isoform X3 gene for gill tissue.

According with the $2^{-\Delta\Delta Ct}$ values, data for each tissue and condition selected were converted to natural logarithm and analysed using a student

T-test (independent). Statistical comparison between the treatment and its respective control were performed, and statistical significances were set at $p < 0.05$. The $\log_2$FC was assessed using treatment vs control data to validate the expression of selected genes compared to the transcriptome results and by a linear regression. The software Statistica® 10.0 (StatSoft, Tulsa, OK, USA) was used to perform statistical analysis.

Results

De novo assembly and functional annotation of the juvenile scallop *Argopecten ventricosus* transcriptome

To evaluate the effect of the immunomodulatory compounds on gene regulation, 20 libraries from juvenile Catarina scallops were considered and treated with the four HDIC (Fig 1B) versus control C1 (Two libraries for experimental condition of each tissue) plus 12 libraries using NexSeq illumine in paired-end mode (2 X 150). A total of 423 million raw pair reads were generated. After the process of filtering reads, 372 million high-quality pair reads were used for the *de novo* transcriptome assembly. The final assembly generated 191 432 transcripts with a maximum length of 36 697 bp, a minimum length of 184 bp, an average length of 863 bp and N50 value of 1123 bp (Fig 2A). The transcript size distribution showed a higher abundance of

transcripts from 100 to 600 bp and a lower abundance of transcripts from 2000 to 2200 bp (Fig 2B). The analysis of transcriptome completeness using BUSCO tool against the database of Metazoa odb9, which consisted of benchmarking universal single-copy of conserved orthologous genes, showed that the percentage of complete and fragmented genes was 79.4% and 17%, respectively (Fig 2D).

Functional annotation results showed that 62 548 (32%) of the transcripts were annotated with at least one database. The *Mizuphecten yesseniosis* genome was the database that allowed the highest number of annotated transcripts (60157 hits). *M. galloprovincialis* genome and KEGG database showed the lowest number of annotated transcripts (15 425 and 17 487, respectively) (Fig 2A). The distribution of the BLAST top hits against the non-redundant protein (NCBI) database showed that the species with the highest number of top hits were the oyster *Crassostrea gigas* (18 339 hits) and the pectinid *Mizuphecten yesseniosis* (12 104 hits) (Fig 2C).

Fig 2. Summary of the *novo* assembled transcriptome of *Argopecten ventricosus juveniles*. (A) Assembly statistics and Functional Annotation of the scallop *A. ventricosus* transcriptome; (B) Size distribution of assembled transcripts; (C) Blast top hit species distribution; (D) BUSCO results. C: complete; CS: complete single-copy; CD: complete duplicate; F: fragmented, and M: missing.

As a result, from the functional annotation of the *A. ventricosus* juvenile transcriptome in Blast2go 47 313 transcripts were annotated. A total of 29 116 transcripts were assigned to some biological processes and the most represented were related to biosynthesis (2749), signal transduction (2 711) and cellular nitrogen compound metabolic process (2485) (Fig 3A). For molecular functions 30 274 transcripts were annotated. The most represented molecular function categories were related to ion binding (6662), oxidoreductase activity (2233) and transmembrane transporter activity (2076) (Fig 3A). In the category of cellular components 19 886 transcripts were recorded. The cellular component with higher number of transcripts corresponded to protein containing complex (3305), cytoplasm (1329) and intracellular (1246) components (Fig 3A). The KEGG annotation mapped 17 487 transcripts and showed the metabolic pathways mostly related to biosynthesis of secondary metabolites (222), thermogenesis (139) and endocytosis (128) (Fig 3B).

Fig 3. Top 25 gene ontology terms and metabolic pathways from *Argopecten ventricosus* juvenile annotation. (A) Histogram of the gene ontology classification showing the top 25 biological process (blue bars), molecular function (yellow bars), and cellular component (purple bars); (B)

Histogram of the top 25 metabolic pathways from the annotation of the *A. ventricosus* juvenile transcriptome using KEGG database (orange bars). Graphics show the number of transcripts that participate in each classification.

Transcriptional response to Highly-diluted immunomodulatory compounds (HDIC)

Mantle was the tissue with the highest DEG number, particularly in organisms from T1 (3933; 2755 up- and 1178 down-regulated). The mantle of scallops treated with T2 (2002; 1021 up- and 981 down-regulated) recorded the lowest DEG number (S2 Table). In gill, the tissue with less DEG, T3 (1744; 664 up- and 1080 down-regulated) recorded the highest DEG number and T1 (1125; 365 up- and 760 down-regulated) the lowest (S2 Table).

The Venn diagram showed that juvenile scallops had a specific response to each treatment as most of the DEG were not shared between treatments. In most of the cases, the mantle recorded a higher DEG number by treatment effect compared to gill. The scallops that recorded the highest number of up- and down-regulated DEG in mantle were from T1/C1 (1841 up, 491 down) while the lowest number of up-regulated DEG were recorded in scallops from T2/C1 (257 up, 275 down) (Fig 4A). The DEG profiles in mantle were clustered, showing that T3 and T4 were clustered in the same group and T1 and T2 in other groups separately (Fig 4B).

In gill, treatments with the highest number of up- and down-regulated DEG were recorded in scallops from T2/C1 (248 up, 309 down) and T3/C1 (235 up, 483 down). The lowest number of up- and down-regulated DEG in gills were recorded in T4/C1 (94 up, 245 down) (Fig 4C). T1 and T2, formulated by *V. parahaemolyticus* and *V. alginolyticus* lysate in different concentration, were clustered in the same group. T3 (*Silicea terra* and Phosphoric acid) and T4 (Vidatox® Habana, Cuba), formulated by commercial brands, were clustered in a different group (Fig 4D).

Fig 4. Venn diagrams of diferentially expressed genes (DEG). Up- and down-regulated DEG in mantle (A) and gill (C) tissue of *Argopecten ventricosus* juveniles treated with highly-diluted immunomodulatory compounds (HDIC). Dendogram shows the relationship between gene expression by hierarchical clustering by treatment in mantle (B) and gill tissue (D). T1: *V. parahaemolyticus* and *V. alginolyticus* lysate 1D; T2: *V. parahaemolyticus* and *V. alginolyticus* lysate 7C; T3: *Silicea terra* and Phosphoric acid 7C; T4: Vidatox® (Habana, Cuba) 31C, and control treatment C1: hydro-alcoholic solution (1%).

Co-regulation of gene expression and functional enrichment analysis

Hierarchical clustering analysis of the differentially expressed transcripts allowed identifying groups of co-expressed transcripts for each tissue and treatment. Furthermore, the GO biological processes and metabolic pathways enriched for each group were analysed (figures 5 and 6). In mantle, the clustering analyses showed 20 clusters; from these clusters seven were enriched by GO terms (*p*-value < 0.01). Interestingly, three groups were related to oxidative phosphorylation (2,12,14) and four groups (cluster 13, 15, 16 and 20) were enriched in biological processes related to non-self-recognition and immune response processes. Cluster 13 comprised five transcripts related to focal adhesion and five related to endocytosis (Fig 5). Most of the DEG from cluster 13 in mantle were down-regulated ($Log_2FC < 0$) mainly by effect of T4/C1 (mean Log_2FC -3.7) (S1 Fig). A total of five transcripts from cluster 15 were related to apoptosis process. DEG from cluster 15 were mainly up-regulated ($Log_2FC > 0$) in all treatments with a higher number of up-regulated DEG in T3/C1 (mean Log_2FC 5.8). In cluster 16, 44 transcripts were related to pathogenic *Escherichia coli* infection pathway, 31 to cytoskeleton organization and eight to immune response. All treatments from cluster 16 in mantle were mainly up-regulated ($Log_2FC > 0$) with the highest number of up-regulated DEG in T1/C1 (mean Log_2FC 2.5) (S1 Fig). Cluster 20 comprised 65 transcripts related to signal transduction and 18 to pathogenic *E. coli* infection pathway. Cluster 20 showed up- ($Log_2FC > 0$) and down- (Log2FC < 0) regulated DEG with the highest number of up-regulated

DEG in T4/C1 (mean Log_2FC 0.7) and the highest number of down-regulated DEG in T1/C1 (mean Log_2FC -0.5) (Fig 5 and S1 Fig). In mantle the enrichment of clustered co-expressed genes were in accordance to the enrichment of all DEG as shown in supporting figures (S2 and S3 Figs).

Fig 5. Heatmap showing clusters of co-regulated genes with their expression profiles in mantle tissue of the Catarina scallop *Argopecten ventricosus* juveniles treated with highly-diluted immunomodulatory compounds (HDIC). The dendrogram shows the relationship between gene expression by hierarchical clustering. Clusters in differentially expressed coloured boxes at the left indicate gene clusters with similar expression profiles. The colour key indicates the Log_2FC of the DEG (FDR < 0.01) ranging from cyan blue for most up-regulated to red for most down-regulated genes. Cluster with functional enrichment with GO or KEGG database with more than five transcripts participating in the process are shown in graph on the right side (C2:Cluster 2, C9:Cluster 9, C10:Cluster 10, C12:Cluster 12, C13:Cluster 13, C14:Cluster 14, C15:Cluster 15, C16:Cluster 16, C20:Cluster 20). Circles show the colour of their corresponding co-regulated gene cluster. Contrast conditions were clustered to identify similarity pattern in the DEG between treatments. T1: *V. parahaemolyticus* and *V. alginolyticus* lysate 1D; T2: *V. parahaemolyticus* and *V. alginolyticus* lysate 7C; T3: *Silicea terra* and Phosphoric acid 7C; T4: Vidatox® (Habana, Cuba) 31C, and control treatment

C1: hydro-alcoholic solution (1%).

On the other hand, 17 groups were generated in gill, of which six showed enriched GO terms (*p*-value < 0.01, Fig 6). The enrichment analysis showed that three groups (6, 7 and 12) were related to oxidative phosphorylation and three (clusters 5, 8 and 16) were related to non-self-recognition and immune response (Fig 6). In cluster 5 from gill tissue, 11 transcripts were related to cellular response to stimulus (Fig 6). Scallops from cluster 5 showed a DEG down-regulation ($Log_2FC < 0$) mainly by effect of T1/C1 (mean Log_2FC -7) (S1 Fig). Cluster 8 comprised 10 transcripts related to NF-kappa B signalling pathway, 44 to signal transduction and 17 to stress response. In cluster 8 the DEG were mainly down-regulated ($Log_2FC > 0$) in all treatments with a higher number of down-regulated DEG in T2/C1 (mean Log_2FC -1). A total of 13 transcripts from cluster 16 were related to transmembrane transport in gill tissue. Transcripts for all the treatments in gill were mainly up-regulated in cluster 16 ($Log_2FC > 0$) with the highest number of up-regulated DEG in T1/C1 (mean Log_2FC 2.5) (Fig 6 and S1 Fig). As recorded in mantle, the enrichment of clustered co-expressed genes from gill tissue were in accordance to the enrichment of all DEG shown in supporting figures (S2 and S3 Figs).

Fig 6. Heatmap showing clusters of co-regulated genes with their

expression profiles in gill tissue of the Catarina scallop *Argopecten ventricosus* juveniles treated with highly-diluted immunomodulatory compounds (HDIC). The dendrogram shows the relationship between gene expressions by hierarchical clustering. Clusters differentially expressed in colour boxes at the left indicate gene clusters with similar expression profiles. The colour key indicates the Log_2FC of the DEG (FDR < 0.01) ranging from cyan blue for most up-regulated to red for most down-regulated genes. Cluster with functional enrichment with GO or KEGG database with more than five transcripts participating in the process are shown in graphs on the right side (C5: Cluster 5, C6: Cluster 6, C7: Cluster 7, C8: Cluster 8, C10: Cluster 10, C12: Cluster 12, C14: Cluster 14, C15: Cluster 15, C16: Cluster 16). Circles show the colour of their corresponding co-regulated gene cluster. Contrast conditions were clustered to identify similarity pattern in the DEG between treatments. T1: *V. parahaemolyticus* and *V. alginolyticus* lysate 1D; T2: *V. parahaemolyticus* and *V. alginolyticus* lysate 7C; T3: *Silicea terra* and Phosphoric acid 7C; T4: Vidatox® (Habana, Cuba) 31C, and control treatment C1: hydro-alcoholic solution (1%).

Identification of genes associated to immune response regulated by effect of highly-diluted immunomodulatory compounds (HDIC)

Since it has been observed that HDIC improves the ability of these organisms to cope with pathogen infections [20], probably through immune system modulation [12,21], the transcripts related to the immune system were identified, and their expression levels were analyzed in response to the treatments. Based on the functional annotation and GO and KEGG categorization, a total of 193 transcripts were identified related to the non-self-recognition receptor, internalisation and immune system. The expression analyses showed that the tissue with higher DEF number related to immune response was mantle (175 transcripts) compared to gill (28 transcripts) (S3 and S4 Table). The more representative metabolic pathways with higher DEG number were phagosome, endocytosis and NF-kappa B signal, but non-self-recognition receptors were also found.

In mantle, higher DEG related to immune response were recorded in scallops from T1(C1 (up: 58, down: 11) and T3/C1 (up: 56 , down: 13), and the lower in T4/C1 (up: 17 , down: 16). The functional analyses of these transcripts showed that the metabolic pathways with higher number of up-regulated DEG in T1/C1 were NF-kappa B signal pathway (10), phagosome (9) and endocytosis (10) in mantle. Also, T1/C1 up-regulated lysosome pathway (4), toll like receptors (1) and heat shock proteins (2) but with a lower DEG number. Mainly down-regulated metabolic pathways in mantle recorded in organisms from T1/C1 were related to the phagosome (6) which also had up-regulated genes. T3/C1 allowed up-regulation of DEG related to endocytosis (up: 8,

down: 3), focal adhesion (7) and phagosome (up: 7, down: 3) in organisms mantle. The highest numbers of down-regulated DEG in T3/C1 were observed in MAPK signalling pathway (4). In scallops from T4/C1, the metabolic pathways with higher number of up-regulated DEG were observed in hematopoietic cell linage (2) and melanogenesis (3) pathway; the highest numbers of down-regulated DEG were recorded in focal adhesion (3) and endocytosis (up: 2 down: 5) pathway (Fig 7 and S3 Table).

In gill, most of the pathways were down-regulated; the treatment with higher DEG was T2/C1 (up: 1, down: 15), and the one with lower DEG was T4/C1 (up: 1, down: 3). As in mantle, scallops from T2/C1 showed a higher number of down-regulated DEG (15) in gill compared with the up-regulated DEG (1). The metabolic pathways with higher number of down-regulated DEG in T2/C1 were recorded in the NF-kappa B signal pathway (4) and endocytosis pathway (up: 1, down: 2). The gill of the organisms treated with T2/C1 up-regulated only one gene, and it was related to endocytosis. The metabolic pathways with higher number of up-regulated DEG in T4/C1 were observed in NOD-like receptor signalling (1) (Fig 7 and S4 Table).

Fig 7. Differentially expressed genes (DEG) modulated for each treatment and tissue (mantle and gill) related to immune response, signal internalisation and non-self-recognition in *Argopecten ventricosus* juveniles treated with highly-diluted immunomodulatory compounds

(HDIC). DEG are grouped in to metabolic pathways categories. T1: *V. parahaemolyticus* and *V. alginolyticus* lysate 1D; T2: *V. parahaemolyticus* and *V. alginolyticus* lysate 7C; T3: *Silicea terra* and Phosphoric acid 7C; T4: Vidatox® 31C, and control treatment C1: hydro-alcoholic solution (1%).

Validation of RNA-seq data by qPCR analysis

Ten relevant DEG related to the immune response and regulated by the four treatments in this study were selected to validate the transcriptome data by RT-qPCR analysis. All contrasts were made using the control group as a reference; relative expression was normalised to the abundance of sodium/potassium-transporting ATPase subunit alpha-like gene in mantle tissue and cAMP-dependent protein kinase catalytic subunit-like isoform X3 gene in gill tissue because they were the most stable genes among the experimental conditions. The up- and down-regulated genes selected from the transcriptome had the same pattern in RT-qPCR analysis. IAP, HSP90; ERIS genes significantly ($p < 0.001$) up-regulated in organism mantle by effect of T1. In T2 gene HSP90 ($p < 0.001$) from the mantle significantly up-regulated and FILA ($p < 0.01$) down-regulated. The genes ERIS, TRAF3 ($p < 0.01$) and SRC ($p < 0.001$) up-regulated in the organism mantle from T3, and in T4 they promoted up-regulation of HEXA gene ($p < 0.001$) (Fig 8A). In gill T1 up-regulated ($p < 0.05$) the expression of TLR2 gene; T2 promoted the

up-regulation ($p < 0.001$) of TRAF3 gene; T3 regulated the expression of TLR3 gene ($p < 0.01$) (Fig 8A). Finally, the correlation between the expression of the genes in mantle and gill using Log_2FC data from RT-qPCR and RNA-seq analyses was 0.80 and 0.94, respectively (Figs 8B and 8C), which were considered good.

Fig 8. Real time qPCR validation of *Argopecten ventricosus* juvenile transcriptome data for selected genes. (A) Relative expression normalized to the abundance of sodium/potassium-transporting ATPase subunit alpha-like gene in mantle tissue and correlation between LogFC transcriptome data and RT-qPCR data in mantle; (B) Relative expression normalised to the abundance of cAMP-dependent protein kinase catalytic subunit-like isoform X3 gene in gill tissue and correlation between LogFC transcriptome data and qPCR data in gill. IAP: Baculoviral IAP repeat-containing protein 3-like, HSP90: Heat shock protein 90, ERIS: Endoplasmic reticulum interferon stimulator, SRC: Src substrate cortactin-like isoform X1, HEXA: Beta-hexosaminidase, CAT: Catalase, TRAF3: TNF receptor-associated factor 3, FILA: Filamin-A-like isoform X3 , TLR2: Toll-like receptor 2, TLR3: Toll-like receptor 3. T1: *V. parahaemolyticus* and *V. alginolyticus* lysate 1D; T2: *V. parahaemolyticus* and *V. alginolyticus* lysate 7C; T3: *Silicea terra* and Phosphoric acid 7C; T4: Vidatox® (Habana, Cuba) 31C, and control treatment C1: hydro-alcoholic solution (1%).

Discussion

Most of the transcriptomes assembled in marine bivalves did not report fragmentation percentage in their results; however, fragmentation is known to be typical in *de novo* transcriptome assemblies [54]. The assembly statistics in this study was according to most published marine bivalve transcriptomes referred by the authors as high quality assembly [55, 56,57] and showed higher quality assembly compared to others [27,58,59].

The effectiveness of the use of HDIC in bivalves to improve their survival against pathogen infection challenges has been widely demonstrated, Our work team has proven that the application of HDIC formulated with scorpion venom, Phosphoric acid, silica, sodium metasilicate and *Vibrio* lysate (*V. alginoliticus*-*V. parahemolityics*) strengthened marine organisms (bivalves, fish and shrimp) self-defence, allowing them to survive infection challenges, reduce parasite proliferation, increase energy reserves and growth; even some of them outperformed antibiotics when they were used prophylactically in *A. ventricosus* culture [20,21,60,61].

The efficiency of these HDIC to protect marine organisms against infections has been attributed to increases of the antioxidant system response, haemocyte count, energetic reserves and enzymes related to food assimilation [20,21,62]. Furthermore, attenuated *V. splendidus* cells activated the immune

response in the scallop *Argopecten purpuratus* [18] and micro-sized silica [63], phosphorus [64] and toxins [65] activating antioxidant response, which allowed organisms to face pathogen infections by promoting free radical eradication. This information considered that the evaluated HDICs may have the potential to modulate immune response at high and low concentrations; however, the mechanisms regulated at low dilutions have not been elucidated yet. Information about how genes and mechanisms are being regulated in marine organisms treated with HDIC is scarce. In this sense, interestingly, the results from the transcriptomic analysis in this study, suggested that the immune response was activated by most of the HDIC but not like conventional immunostimulants, such as polysaccharides, nutrients, oligosaccharides, herbs, antibacterial peptides and microorganisms [11] used at higher concentrations, which increased production of bactericidal and cytotoxicity activities, lysozyme and antimicrobial peptides [11,66,67]. In this study, HDIC regulated transcripts associated to recognition of non-self-molecules and internalisation of particles suggested that HDIC were not able to trigger all the immune response mechanisms due their high dilution. This result may explain those [60] in organisms of *S. rivoliana* treated with HDIC where only the genes IL-1β and MyD88 up-regulate before the challenge with *V. parahaemolyticus*. The results in this study suggested that the non-self-recognition system activation by effect of HDIC improved the response against infections. When the defence mechanisms were activated before the infection process, the

organisms had more probabilities to successfully overcome a disease, which was the principle of the immunostimulants used in aquaculture [11].

It is worth to highlight that most of the DEG related to non-self-recognition, internalisation and immune response were detected in mantle compared to gill tissue. This result can be explained because mantle and mucosal epithelial cells are one of the first barriers that are in contact with the environment, and epithelial cells have been recognised as the first line of defence against organic, inorganic and pathogen intruders [68]. Epithelial cells are able to endocytose biotic and abiotic particles and activate signals to proliferate haemocytes, the principal effector of the immune response [69], which in turn can also eliminate intruders by endocytosis and enhance immune response [69]. Both epithelial cells and haemocytes can detect intruders by many pattern recognition receptors (PRR) that allow organisms to detect potential danger signals [68]. Thus, the up-regulated transcripts related to non-self-recognition, internalisation and immune response in mantle may be due to the participation of epithelial mantle cell, migrating haemocytes or both. Future research should clarify and corroborate which cells are interacting and recognising the HDIC.

The recognition mechanisms of marine bivalves that detect non-self-molecules are integrated by the PRR, which are activated by pathogen-associated molecular patterns (PAMPs), endogenous ligands (e.g. HSP70) and damage associated molecular patterns (DAMPs). The activation

of the PRR allow organisms to activate the immune response depending on the stimuli received [58]. In agreement with the previous information, the results in this study showed that the up-regulated transcripts are related to PRR from the toll-like receptors family, which are highly conserved in the animal kingdom and have proven to activate the immune response in marine bivalves [58]. Specifically, this study detected the induction of TLR2 by the effect of T1 and T2, and TLR4 in T2 and T3. Moreover, T3 up-regulated TLR3. The activation of TLR2 and TLR4 in T1 and T2 was attributed to their formulation (*V. alginoliticus* and *V. parahemolityics*) because TLR2 and TLR4 are Pattern recognition receptors (PRR) located in plasma membrane and mainly recognise bacterial PAMPs, such as lipopolysaccharides [70] that can be found in gram negative bacteria like *V. alginoliticus* and *V. parahemolityics* [71]. In bibliography no data was found between the interaction of TLR3 and *Silicea terra* or Phosphoric acid, which were the components in T3, but we only know that TLR3 is a cytosolic receptor that detects non-self-nucleic acids [72]. In this sense, this study reports for the first time that TLR3 is associated to the exposure of highly-diluted *Silicea terra* or Phosphoric acid. Additionally, these results showed that T3 formulated by *Silicea terra* and Phosphoric acid promoted the induction of the TLR4 gene. Accordingly, evidence that SiO_2 nanoparticles are able to increase the TLR4a gene expression has been observed in Dino zebrafish embryos [73], and *S. rivoliana* juveniles treated with *Silicea terra* - Phosphoric acid up-regulated MyD88 transcript only when

organism were challenged. It should be noted that MyD88 regulation is highly related to TLR4 [74], which suggested that the up-regulation of TLR4 in T3 was associated to *Silicea terra* action. In T4, formulated with Vidatox® (Habana, Cuba), no PRR regulated were observed, which may explain the low regulation of transcripts related to immune response. The information above suggested that HDIC had been detected mainly through toll-like receptors in treatments T1, T2 and T3, while Vidatox® (Habana, Cuba) may have detected them by an alternative route in T4.

When small intruders (e.g. Bacteria, LPS) are detected via PRR, many mechanisms can be activated to respond against the potential danger signals, such as PAMPs and DAMPs [69]. The mechanisms activated in this study were dependent on each treatment and their dilutions. Treatments T1 and T2 mainly modulated the expression of transcripts related to endocytosis, lysosome, phagosome, proteasome and NF-kappa B pathways. Interestingly, T1, which had a higher concentration of bacterial compounds than T2, up-regulated transcripts associated to those processes while T2 regulated them negatively. Other authors have reported that the presence of PAMPs activated endocytosis, lysosome, and phagosome mechanisms [68,69] to destroy potentially harmful molecules or disease-causing microorganisms. The activation of those mechanisms can also activate the signalling cascade to express immune related genes [68,69] via NF-kappa B pathway signal [75]. This result suggested that the organisms treated with T1 recognised and

responded to the presence of PAMPs or DAMPs in the HDIC formulated by *V. parahaemolyticus* and *V. alginolyticus* lysate.

In this study, most of the transcripts that up-regulated in NF-kappa B pathway by T1 were modulators at the first level of the immune response and effectors of the anti-apoptosis process. In this sense, T1 allowed activating a conservative response. The results showed that T1 also up-regulated genes related to sense microbial viability (STING), which allowed organisms to detect living bacteria [76], bind Vibrio protein (CALR) that can be released from the cell and neutralise vibrio [77,78] and heat shock proteins (HSP70 and HSP90), related to showing antigen (adaptive immune system) [79], protein fold [80], activating Toll-like receptors [81] and mediating immune response in bacterial challenges [82]. The above suggested that T1 allowed the activation of pathways related to recognition, neutralisation, internalisation and destruction of *Vibrio*. Although T2 did not activate transcripts associated to mechanisms of endocytosis, lysosome, phagosome, proteasome and NF-kappa B pathways, it has been successfully used in strengthening marine organisms against pathogens [12,13,60]. These results showed that the induction of TLRs in scallops by T2 may have been enough to protect organisms against pathogens. Because T2 was highly diluted compared to T1, the concentration of molecules from the *V. parahaemolyticus* and *V. alginolyticus* lysate might have not been enough to activate the internalisation and destruction of non-self-molecule mechanisms allowing organisms to tolerate the presence of these molecules

and increase the expression of the transcripts related to detection mechanisms of potential danger signals, such as TLRs, which may imply a lower energetic cost for the organisms. Perhaps, as it was reported, PAMPs used at high-doses induced tolerance while at lower ones it allowed trained sensitization, and in very low doses, it did not activate immune response [83]. Higher dilutions from the HDIC used in this study were in accordance with literature [83], which proposed that very low doses of PAMPs did not activate innate immune response. However, for the first time in *Argopecten ventricosus* this study detected the up-regulation of transcripts related to TLRs without activating the immune response. Contrary to very low doses, the low doses of HDIC in this study showed an activation of detection, neutralisation and internalisation responses against intruders without activating all the immune response mechanisms. The above suggests that a prolonged exposure time to low PAMPs doses may also generate some tolerance because the activation of immune response implies high ATP amounts. The reason why innate cells depend on PAMPs dose and exposure time may be explained by the energetic cost; if organisms sense a very low or low concentrations of PAMPs, the energetic balance is maintained; the organisms can invest energy in processes related to self-protection depending on the dose and time of exposure to the stressor (e.g. LPS).

Interestingly, organisms treated with T3 (*Silicea terra* - Phosphoric acid) induced transcripts related to bacterial challenge (STING, CALR), antioxidant

system (CAT, PRDX6) [58,84], cell migration (CTTN) [85], amplification of immune response (CD40LG, found in adaptive response) [86], and the receptor associated to actin-assembly machinery on the cytoplasmic side of the phagosome (P2X7) [87]. In previous publications, *Silicea terra* and Phosphoric acid had recorded an improvement of *A. ventricosus* juvenile growth, antioxidant activity [21] and survival when organisms were challenged against pathogenic bacteria [20]. Moreover, silicic acid, which has been reported in silica solutions [88], may activate catalase (CAT) and superoxide dismutase (SOD) activities in mouse brain from organisms intoxicated with aluminium [89]; in *S. rivoliana*, the use of S*ilicea terra* and Phosphoric acid increases survival when organisms are challenged against pathogenic bacteria [60]. These results suggested that the activation of non-self-recognition, endocytosis and antioxidant system by T3 was the reason for survival when organisms were challenged.

As mentioned before, T4 behaved differently compared to the rest of the treatments and mainly activated the melanogenesis and haematopoietic processes and a gene associated to calcium transport (TRPM2), which have been linked to immune response when organisms are challenged against bacteria [69,70]; however, the immunomodulatory effect of Vidatox® (Habana, Cuba) was not clear. Vidatox® (Habana, Cuba) has demonstrated to increase SOD activity and survival of *L. vannamei* when it has been challenged against pathogenic bacteria [90], but it has also been related to proliferation of

hepatocellular carcinoma in cultured mouse cells [91]. As in the other treatments, oxidative phosphorylation in mitochondria was modulated by the effect of T4. The activation of the immune response may be modulated by alternative routes as inflammasomes that are activated by DAMPs [70] and highly related to the production of mitochondria ROS (mROS), which have been implied in the activation of the immune response [70,87]. This result could explain the hepatocellular carcinoma proliferation in cultured mouse cell when Vidatox® (Habana, Cuba) was used since proliferation of mROS may cause oxidation damage in cancer cells [92]; the positive effect recorded in challenged shrimp, as mROS may also have been related to the activation of immune response and killing pathogens [70]. Thus, T4 may be more related to the activation of immune response by mitochondria signalling. Interestingly, T4 was formulated by the most diluted compound, suggesting that molecules highly diluted mainly act by their magnetic charge [14], sensed by traditional molecular receptors or photoelectrochemical sensing system activated by ultra-weak photo emission signals [15]. In *Arabidopsis thaliana* organisms their defence mechanisms may be activated by the sound of caterpillars (*Pieris rapae*) eating their leaves [93] *via* mitochondria signalling; their mROS production has been implied in vibration recognition [94]. Since mitochondria have been related to the activation of the immune response by vibration recognition mechanisms, future research should analyse the effect of Vidatox® (Habana, Cuba), taking into account the importance of mitochondria and

oxidative phosphorylation process.

It is worth to notice that all the treatments modulated the oxidative phosphorylation process in mitochondria, an organelle that is not only related to vibration recognition but also plays a crucial role in supplying ATP since it is necessary for cell internalisation process, acidification or the phagosome, production of mROS for eradication of bacteria and activation of immune signalling cascades [70,87]. Nonetheless, the up- or down-regulated process could not be detected due to transcriptome fragmentation (17%), which diminished the robustness of expression estimates in fragmented genes [54].

This study used different HDIC, most of them showing up-regulation in the PAMPs recognition system, which may explain why marine organisms increased survival challenges against pathogens in previous studies [12].

Conclusion

This study opens new insight into the activation of *Argopecten ventricosus* self-protection mechanism using HDIC, which allowed answering questions and opening the possibility of generating others about interactions between organisms and their exposure to low toxin concentration, contaminants, minerals, pathogens and other stress factors during a prolonged time. The results of this study implied that lower dilutions activated mechanisms of immune response in a higher level in a shorter period of time

while higher dilutions activated only the first defence mechanisms in *A. ventricosus*, which may be dependent on the exposure time between the organisms and the HDIC. These results showed the complex dynamics between the non-self-recognition mechanisms of *A. ventricosus* juveniles and HDIC in a long time exposure. Additionally, mitochondria, besides its role in energy production, may play an important role in defence response activation and signalling process when organisms are exposed to HDIC. Furthermore, this is the first assembled transcriptome of the scallop *A. ventricosus* juveniles using RNA-seq technology, which allows us to generate biological markers for future investigation of this important resource whose populations are declining in Baja California Sur, México.

Acknowledgements

This study was funded by the Sectoral Fund for Research for Education of México; projects Basic Science CONACyT 258282 and PROINNOVA - CONACyT 24177 under the academic responsibility of JMMS. JALC is the recipient of a doctorate fellowship (CONACYT-301921), under the academic direction of FAO and JMMS. They conceived and assessed the experimental design and research development and JALC performed zoo-technical work, collected and analysed data, and wrote the first draft of the manuscript. All authors revised and approved the final manuscript. The authors are grateful to

the technical staff at CIBNOR: Delfino Barajas, Pablo Ormart, Julián Garzón, Carmen Rodríguez, Patricia Hinojosa, Norma Ochoa, Martín Ramírez, Martha Reyes and Eulalia Meza for their support. Diana Fischer provided editorial services in English. Noroeste Sustentable A.C. donated scallops produced by Acuacultura Robles hatchery.

References

1. Travers MA, Miller, Roque A, Frideman CS. Bacterial diseases in marine bivalves. J Invertebr Pathol. 2015;131: 11-31. doi: 10.1016/j.jip.2015.07.010.

2. Kesarcodi-Watson A, Kaspar H, Lategan MJ, Gibson L. Probiotics in aquaculture: the need, principles, and mechanisms of action and screening processes. Aquaculture. 2008;274: 1-14. doi: 10.1016/j.aquaculture.2007.11.019.

3. Dubert J, Barja, JL, Romalde JL. New insights into pathogenic Vibrios affecting bivalves in hatcheries: present and future prospects. Front Microbiol. 2017;8: 1-16. doi: 10.3389/fmicb.2017.00762.

4. Wendling CC, Batista FM, Wegner KM. Persistence, seasonal dynamics and pathogenic potential of *Vibrio* communities from Pacific oyster hemolymph. PLoS ONE. 2014;9: e94256. doi:

10.1371/journal.pone.0094256.

5. Semenza JC, Trinanes J, Lohr W, Sudare B, Löfdahl M, Martinez-Urtaza J, et al. Environmental suitability of Vibrio infections in a warming climate: an early warning system. Environ Health Perspect. 2017;125: 107004-1-107004-12. doi: 10.1289/ehp2198.

6. Prado S, Romalde JL, Barja JL. Review of probiotics for use in bivalves hatcheries. Vet Microbiol. 2010;145: 187-197. doi: 10.1016/j.vetmic.2010.08.021.

7. Romero J, Feijo CG, Navarrete P. Antibiotics in aquaculture – use, abuse, and alternatives. In: Carvalho E, editor. Health and environmental aquaculture. In Tech, Rijeka; 2012. pp. 159-198.

8. Liu X, Steele JC, Meng XZ. Usage, residue, and human health risk of antibiotics in Chinese aquaculture: A review. Environ Pollut. 2017;223: 161-169. doi: 10.1016/j.envpol.2017.01.003.

9. Liang P, Wu S, Zhang C, Xu J, Christine P, Zhang J, et al. The role of antibiotics in mercury methylation in marine sediments. J Hazard Mater. 2018;360: 1-5. doi: 10.1016/j.jhazmat.2018.07.096.

10. Lawrence R, Jeyakumar E. Antimicrobial Resistance: A Cause for Global Concern. BMC Proc. 2013;7, S1. doi: 10.1186/1753-6561-7-S3-S1.

11. Wang W, Sun J, Liu C, Xue Z. Application of immunestimulants in

aquaculture: current knowledge and future perspectives. Aquac Res. 2017;48: 1-23. doi: 10.1111/are.13161.

12. Ortiz-Cornejo NL, Tovar-Ramírez D, Abasolo-Pacheco F, Mazón-Suástegui JM. Homeopatía, una alternativa para la acuicultura. Revista Médica de Homeopatía. 2017;10: 28-34. doi: 10.1016/j.homeo.2017.04.006.

13. Mazón-Suástegui JM, García-Bernal M, Avilés-Quevedo A, Campa-Córdova A, Salas-Leiva J, Abasolo-Pacheco F. Assessment of homeopathic medicines on survival and antioixidant response in white shrimp *Litopenaeus vannamei*. rev MVZ Cordoba. 2018;23(3): 6850-6859. doi: 10.21897/rmvz.1373.

14. Bellavite P, Marzotto M, Olioso D, Moratti E, Conforti A. High-dilution effects revisited. 1. Physicochemical aspects. Homeopathy. 2014;103: 4-21. doi: 10.1016/j.homp.2013.08.003.

15. Prasad A, Rossi C, Lamponi S, Pospíšil P, Foletti A. New perspective in cell communication: potential role of ultra-weak photon emission. J Photochem Photobiol. 2014;139: 47–53. doi: 10.1016/j.jphotobiol.2014.03.004.

16. Bell IR, Sarter B, Koithan M, Standish LJ, Banerji P, Banerji P. Nonlinear response amplification mechanisms for low doses of natural product

nanomedicines: dynamical Interactions with the recipient complex adaptive system. J Nanomed Nanotechnol. 2013;4: 179. doi: 10.4172/2157-7439.1000179.

17. Bell IR, Schwartz GE. Enhancement of adaptive biological effects by nanotechnology preparation methods in homeopathic medicines. Homeopathy, 2015;104: 123-138. doi: 10.1016/j.homp.2014.11.003.

18. González R, Brokordt K, Cárcamo CB, Coba de la Peña T, Oyanedel D, Mercado L, et al. Molecular characterization and protein localization of the antimicrobial peptide big defensing from the scallop *Argopecten purpuratus* after *Vibrio splendidus* challenge. Fish Shellfish Immunol. 2017;68: 173-179. doi: 10.1016/j.fsi.2017.07.010.

19. Pauletto M, Milan M, Moreira R, Novoa B, Figueras A, Babbucci M, et al. Deep transcriptome sequencing of *Pecten maximus* hemocytes: a genomic resource for bivalve immunology. Fish Shellfish Immun. 2014;37: 154-165. doi: 10.1016/j.fsi.2014.01.017.

20. Mazón-Suástegui JM, García-Bernal M, Saucedo PE, Campa-Córdova A, Abasolo-Pacheco F. Homeopathy outperforms antibiotics treatments in juvenile scallop *Argopecten ventricosus*: effects on growth, survival, and immune response. Homeopathy. 2017;106(1): 18-26. doi: 10.1016/j.homp.2016.12.002.

21. López-Carvallo JA, Arcos-Ortega GF, Tovar-Ramírez D, Hernández-Oñate MA, Abasolo-Pacheco F, García-Corona JL, et al. Effect of immunomodulatory medication over the general response of juvenile Catarina scallop (*Argopecten ventricosus* Sowerby II, 1842). Lat Am J Aquat Res. 2019;47(1): 65-77. doi: 10.3856/vol47-issue1-fulltext-8.

22. Hu YF, Kaplow J, He Y. From Traditional Biomarkers to Transcriptome Analysis in Drug Development. Curr Mol Med. 2005;5: 29-38. doi: 10.2174/1566524053152915.

23. Nie H, Jiang L, Chen P, Huo Z, Yang F, Yan X. High throughput sequencing of RNA transcriptomes in *Ruditapes philippinarum* identifies genes involved in osmotic stress response. Sci Rep. 2017;7:4953. doi: 10.1038/s41598-017-05397-8.

24. Kim BM, Kim K, Choi IK, Rhee JS. Transcriptome response of the Pacific oyster, *Crassostrea gigas* susceptible to thermal stress: A comparison with the response of tolerant oyster. Mol Cell Toxicol. 2017;13: 105-113. doi: 10.1007/s13273-017-0011-z.

25. Mat AM, Klopp C, Payton L, Jeziorski C, Chalopin M, Amzil Z, et al. Oyster transcriptome response to *Alexandrium* exposure is related to saxiotoxin load and characterized by disrupted digestion, energy balance, and calcium and sodium signaling. Aquat Toxicol. 2018;199: 127-137. doi: 10.1016/j.aquatox.2018.03.030.

26. Shi P, Dong S, Zhang H, Wang P, Niu Z, Fang Y. Transcriptome profiling analysis of *Mactra veneriformis* by deep sequencing after exposure to 2,2´,4,4´-tetrabromodiphenyl ether. J Ocean Limnol. 2018;36: 490-507. doi: https://doi.org/10.1007/s00343-018-6347-y.

27. Moreira R, Balseiro P, Forn-Cuni G, Milan M, Bargelloni L, Novoa B, et al. Bivalve transcriptomic reveals pathogen sequences and a powerful immune response of the Mediterranean mussel (*Mytilus galloprovincialis*). Mar Biol. 2018;165: 61. doi: 10.1007/s00227-018-3308-0.

28. Gómez-Chiarri M, Guo X, Tanguy A, He Y, Proestou D. The use of -omics tools in the study of disease processes in marine bivalves mollusks. J Invertebr Pathol. 2015;131: 137-154. doi: 10.1016/j.jip.2015.05.007.

29. Carta Nacional Pesquera. Diario oficial de la federación. México, D.F. 2018 Jun 11 [cited 2019 Nov 29]. Available from: https://www.gob.mx/cms/uploads/attachment/file/334832/DOF_-_CNP_2017.pdf

30. Luna-González A, Maeda-Martínez AN, Sainz JC, Ascencio-Valle, F. Comparative susceptibility of veliger larvae of four bivalve mollusks to *Vibrio alginolyticus* strain. Dis Aquat Organ. 2002;49: 221-226. doi: 10.3354/dao049221

31. Bachère E, Rosa RD, Schmitt P, Poirier AC, Merou N, Charrière GM, et al.

The new insights into the oyster antimicrobial defense: Cellular, molecular and genetic view. Fish Shellfish Immun. 2015;46(1): 50–64. doi: 10.1016/j.fsi.2015.02.040

32. Gerdol M, Venier P. An updated molecular basis for mussel immunity. Fish Shellfish Immun. 2017;46(1): 17-38. doi: 10.1016/j.fsi.2015.02.013.

33. Mazón-Suástegui JM, Tovar-Ramírez D, Ortiz-Cornejo NL, García-Bernal M, López-Carvallo JA, Salas-Leiva JS, et al. Effect of homeopathic medicines on growth, survival and gastrointestinal microbiota of juvenile scallop *Argopecten ventricosus*. Rev mvz Córdoba. 2019;24(3): 7328-7338. doi: 10.21897/rmvz.1536.

34. Morelos RM, Ramírez JL, García-Gasca A, Ibarra AM. Expression of the Myostatin Gene in the Adductor Muscle of the Pacific Lion-Paw Scallop *Nodipecten subnodosus* in Association With Growth and Environmental Conditions. J Exp Zool. 2015;323A: 239-255. doi: 10.1002/jez.1914

35. Andrews, S. FastQC: a quality control tool for high throughput sequence data. 2018 Oct 1 [cited 2019 Nov 29]. In: Babraham Institute [Internet]. Available from: http://www.bioinformatics.babraham.ac.uk/projects/fastqc

36. Bolger AM, Lohse M, Usadel B. Trimmomatic: A flexible trimmer for Illumina Sequence Data. Bioinformatics. 2014;30: 2114-2120. doi: 10.1093/bioinformatics/btu170.

37. Haas BJ, Papanicolaou A, Yassour M, Grabherr M, Blood PD, Bowden J, et al. *De novo* transcript sequence reconstruction from RNA-seq using the Trinity platform for reference generation and analysis. Nat Protoc. 2013;8(8): 1494-512. doi: 10.1038/nprot.2013.084.

38. Li B, Dewey CN. RSEM: accurate transcript quantification from RNA-seq data with or without a reference genome. BMC Bioinformatics. 2011;12: 323. doi: 10.1186/1471-2105-12-323.

39. SeqClean website. Available from: https://sourceforge.net/projects/seqclean/. Accessed: 2019 Nov 29

40. Limin F, Beifang N, Zhengwei Z, Sitao W, Weizhong L. CD-HIT: accelerated for clustering the next generation sequencing data. Bioinformatics. 2012;28(23): 3150-3152. doi: 10.1093/bioinformatics/bts565.

41. Zheng Y, Zhao L, Gao J, Fei Z. iAssembler: a package for de novo assembly of Roche-454/Sanger transcriptome sequences. BMC Bioinformatics. 2011;12: 453. doi: 10.1186/1471-2105-12-453.

42. Schmieder R, Edwards R. Quality control and preprocessing of metagenomic datasets. Bioinformatics. 2011;27: 863-864. doi: 10.1093/bioinformatics/btr026.

43. Smith-Unna RD, Boursnell C, Patro R, Hibberd JM, Kelly S. TransRate:

reference free quality assessment of *de-novo* transcriptome assemblies. Genome Res. 2016;26: 1134-1144. doi: 10.1101/gr.196469.115.

44. Waterhouse RM, Seppey M, Simão FA, Manni M, Ioannidis P, Klioutchnikov G, et al. BUSCO Applications from Quality Assessments to Gene Prediction and Phylogenomics. Mol Biol Evol. 2017;35(3): 543-548. doi: 10.1093/molbev/msx319.

45. Götz S, García-Gómez JM, Terol J, Williams TD, Nagaraj SH, Nueda MJ, et al. High-throughput functional annotation and data mining with the Blast2GO. Nucleic Acids Res. 2008;36(10): 3420-3435. doi: 10.1093/nar/gkn176.

46. Chen Y, Lun ATL, McCarthy DJ, Ritchie ME, Phipson B, Hu Y, et al. Package ‘edgeR’. 2019 Dec 10. Available from: https://bioconductor.org/packages/release/bioc/manuals/edgeR/man/edgeR.pdf.

47. Xie C, Mao X, Huang J, Ding Y, Wu J, Dong S, et al. KOBAS2.0: a web server for annotation and identification of enriched pathways and diseases. Nucleic Acids Res. 2011;39: W316-W322. doi: 10.1093/nar/gkr483.

48. Warnes GR, Bolker B, Bonebakker L, Gentleman R, Liaw WHA, Lumely T, et al. gplots: Various R Programming Tools for Plotting Data. 2019 Jan 21. Available from: http://cran.r-project.org/web/packages/gplots/index.html.

49. Untergasser A, Cutcutache I, Koressaar T, Ye J, Faircloth BC, Remm M, et al. Primer3 - new capabilities and interfaces. Nucleic Acids Res. 2012;40(15): e115. doi: 10.1093/nar/gks596.

50. OligoAnalyzer Tool. Available from: https://www.idtdna.com/pages/tools/oligoanalyzer. Accessed: 2019 Nov 29.

51. Xie F, Peng X, Chen D, Lei X, Zhang B. miRDeepFinder: a miRNA analysis tool for deep sequencing of plant small RNAs. Plant Mol Biol. 2012;80: 75-84. doi: 10.1007/s11103-012-9885-2.

52. Bustin SA, Benes V, Garson JA, Hellemans J, Huggett J, Kubista M, et al. The MIQE guidelines: minimum information for publication of quantitative real-time PCR experiments. Clin Chem. 2009;55(4): 611–22. doi: https://doi.org/10.1373/clinchem.2008.112797

53. Livak KJ, Schmittgen TD. Analysis of Relative Gene Expression Data Using Real-Time Quantitative PCR and the $2^{-\Delta\Delta Ct}$ Method. Methods. 2001;25: 402-408. doi: 10.1006/meth.2001.1262.

54. Freedman AH, Clamp M, Sackton TB. Error, noise and bias in *de novo* transcriptome assemblies. BioRxiv [Preprint]. 2019 bioRxiv 585745 [posted 2019 Aug 20; cited 2019 Nov 14]: [44 p.]. Available from: https://www.biorxiv.org/content/10.1101/585745v4 doi: 10.1101/585745

55. Dong W, Chen Y, Lu W, Wu B, Qi P. Transcriptome analysis *Mytilus coruscus* hemocytes in response to *Vibrio alginnolyficus* infection. Fish Shellfish Immun. 2017;70: 560-567. Doi: 10.1016/j.fsi.2017.08.034.

56. Ren Y, Xue J, Yang H, Pan B, Bu W. Transcritpome analysis of *Ruditapes philippinarum* hepatopancreas provides insights into immune signaling pathways under *Vibrio anguillarum* infection. Fish Shellfish Immun. 2017;64: 14-23. doi: 10.1016/j.fsi.2017.03.005.

57. Ventoso P, Pazos AJ, Pérez-Parallé ML, Blanco J, Triviño JC, Sánchez JL. RNA-seq Transcriptome Profiling of the Queen Scallop (*Aequipecten opercularis*) Digestive Gland after Exposure to Domic Acid-Producing *Pseudo-nitzschia*. Toxins. 2019;11: 19. doi: 10.3390/toxins11020097.

58. Ertl NG, O'Connor WA, Papanicolaou A, Wiegand AN, Elizur A. Transcriptome Analysis of the Sydney Rock Oyster, *Saccostrea glomerata*: Insights into Molluscan Immunity. PLoS ONE. 2016;11(6): 1-33. doi: 10.1371/journal.pone.0156649.

59. Zhao X, Duan X, Wang Z, Zhang W, Li Y, Jin C, et al. Comparative transcriptome analysis of *Sinonovacula constricta* in gills and hepatopancreas in response to *Vibrio parahaemolyticus* infection. Fish Shellfish Immun. 2017;67: 523-535. doi: 10.1016/j.fsi.2017.06.040.

60. Mazón-Suástegui JM, Salas-Leiva J, Teles A, Tovar-Ramírez D. Immune and Antioixidant Enzyme Response of Longfin Yellowtail (*Seriola rivoliana*) Juveniles to Ultra-diluted Substances Derived from Phosphorus, Silica and Pathogenic Vibrio. Homeopathy. 2019;108(1): 43-53. doi: 10.1055/s-0038-1672197.

61. Rosero-García AP, Mazón-Suástegui JM, Dumas S, Chávez-Sánchez MC, Avilés-Quevedo A, Rodríguez-Jaramillo C. Effect of Homeopathic Medicines on Intestinal Coccida and Immune Response Cell in Spotted Rose Snapper (*Lutjanus guttatus*). Homeopathy. 2019;108(3): 201-213. doi: 10.1055/s-0039-1681062.

62. Mazón-Suástegui JM, Salas-Leiva J, Teles A, Tovar-Ramírez D. Evaluation of Homeopathic Phosphoric Acid, Silica and Pathogenic *Vibrio* on Digestive Enzymes Activity of Longfin Yellowtail Fish (*Seriola rivoliana*). Homeopathy. 2019;108: 43. doi: 10.1055/s-0039-1692998.

63. Phromkunthong W. Effect of silica supplement on growth performance and health condition of juvenile shrimp. Aqua Asia Pac. 2015;11: 43-46.

64. Jin JL, Wang CF, Tang Q, Xie CX, Dai ZG. Dietary phosphorus affected growth performance, body composition, antioxidant status and total P discharge of young hybrid sturgeon (♀*Huso huso* × ♂*Acipenser schrenckii*) in winter months. J Appl Ichthyol. 2012;28: 697-703. doi: 10.1111/j.1439-0426.2012.02024.x.

65. Prego-Faraldo MV, Vieira LR, Eirin-Lopez JM, Méndez J, Guilhermino L. Transcriptional and biochemical analysis of antioxidant enzymes in the mussel *Mytilus galloprovincialis* during experimental exposures to the toxic dinoflagellate *Prorocentrum lima*. Mar Environ Res. 2017;129: 304-315. doi: 10.1016/j.marenvres.2017.06.009.

66. Antony SP, Singh SB, Sudheer NS, Vrinda S, Priyaja P, Philip R. Molecular characterization of a crustin-like antimicrobial peptide in the giant tiger shrimp, *Penaeus monodon*, and its expression profile in response to various immunostimulants and challenge with WSSV. Immunobiology. 2011;216(1-2): 184-194. doi: 10.1016/j.imbio.2010.05.030.

67. Mohan K, Ravichandrana S, Muralisankarb T, Uthayakumarc V, Chandirasekarc R, Seedevid P, et al. Application of marine-derived polysaccharides as immunostimulants in aquaculture: A review of current knowledge and further perspectives. Fish Shellfish Immun. 2019;86: 1177-1193. doi: 10.1016/j.fsi.2018.12.072.

68. Günther J, Seyfert HM. The first line of defence: insights into mechanisms and relevance of phagocytosis in epithelial cells. Semin Immunol. 2018;40: 555-565. doi: 10.1007/s00281-018-0701-1

69. Allam B, Raftos D. Immune responses to infectious diseases in bivalves. J Invertebr Pathol. 2015;131: 121–36. doi: 10.1016/j.jip.2015.05.005

70. West AP, Shadel GS, Ghosh S. Mitochondria in innate immune responses. Nat Rev Immunol. 2011;11: 389-402. doi: 10.1038/nri2975.

71. Hisatsune K, Kondo S, Iguchi T, Machida M, Asou S, Inaguma M, et al. Sugar Composition of Lipopolysaccharides of Family *Vibrionaceae* Absence of 2-keto-3-deoxyoctonate (KDO) except in *Vibrio parahaemolyticus* O6. Microbiol Immunol. 1982;26(8): 649-664. doi: 10.1111/j.1348-0421.1982.tb00209.x.

72. Tatematsu M, Nishikawa F, Seya T, Matsumoto M. Toll-like receptor 3 recognizes incomplete stem structures in single-stranded viral RNA. Nat commun. 2013;4: 1833. doi: 10.1038/ncomms2857

73. Hu H, Li Q, Jiang L, Zou Y, Duan J, Sun Z. Genome-wide transcriptome analysis of silica nanoparticles-induced toxicity in zebra embryos. Toxicol Res. 2016;5(2): 609-620. doi: 10.1039/c5tx00383k.

74. Laird MH, Rhee SH, Perkins DJ, Medvedev AE, Piao W, Fenton MJ, et al. TLR4/MyD88/PI3K interactions regulate TLR4. J Leukoc Biol. 2009;85(6): 966-977. doi: 10.1189/jlb.1208763.

75. Genard B, Miner P, Nicolas JL, Moraga D, Boudry P, Pernet F, et al. Integrative study of physiological changes associated with bacterial infection in Pacific oyster larvae. PLoS ONE. 2013;8(5): e64534. https://doi.org/10.1371/journal.pone.0064534

76. Moretti J, Roy S, Bozec D, Martinez J, Chapman JR, Uberheide B, et al. STING Senses Microbial Viability to Orchestrate Stress-Mediated Autophagy of the Endoplasmic Reticulum. Cell. 2017;171(4): 809-823.e13. doi: 10.1016/j.cell.2017.09.034.

77. Tarr JM, Young PJ, Morse R, Shaw DJ, Haigh R, Petrov PG, et al. A mechanism of release of calreticulin from cells during apoptosis. J Mol Biol. 2010;401(5): 799-812. doi: 10.1016/j.jmb.2010.06.064.

78. Huang Y, Hui K, Jin M, Yin S, Wang W, Ren Q. Two endoplasmic reticulum proteins (calnexin and calreticulin) are involved in innate immunity in Chinese mitten crab (*Eriocheir sinensis*). Sci Rep. 2016;6: 27578. doi: 10.1038/srep27578.

79. Robert RJ, Agius C, Saliba C, Bossier P, Sung YY. Heat shock proteins (chaperones) in fish and shellfish and their potential role in relation to fish health: a review. J Fish Dis. 2010;33: 789-801. doi: 10.1111/j.1365-2761.2010.01183.x

80. Iryani MTM, MacRace TH, Panchakshari S, Tan J, Bossier P, Wahid MEA, et al. Knockdown of heat shock protein 70 (Hsp70) by RNAi reduces the tolerance of *Artemia franciscana* nauplii to heat and bacterial infection. J Exp Mar Biol Ecol. 2017;487: 106-112. doi: 10.1016/j.jembe.2016.12.004.

81. Asea A, Rehli M, Kabingu E, Boch JA, Baré O, Auron PE, et al. Novel

Signal Transduction Pathway Utilized by Extracelular HSP70. J Biol Chem. 2002;277(17): 15028-15034. doi: 10.1074/jbc.m200497200.

82. Yue X, Liu B, Sun L, Tang B. CLoning and characterization of hsp70 from Asiatic hard clam Metrix metrix which is involved in the immune response against bacterial infection. Fish Shellfish Immun. 2011;30(3): 791-799. doi: https://doi.org/10.1016/j.fsi.2010.12.027.

83. Bauer M, Weis S, Netea MG, Wetzker R. Remembering Pathogen Dose: Long-Term Adaptation in Innate Immunity. Trends Immunol. 2018;39: 438-445. doi: 10.1016/j.it.2018.04.001.

84. Genard B, Moraga D, Pernet F, David É, Boundry P, Tremblay R. Expression of candidates genes related to metabolism, immunity and cellular stress during massive mortality in American oyster *Crassostrea virginica* larvae in relation to biochemical and physiological parameters. Gene. 2012;499(1): 70-75. doi: 10.1016/j.gene.2012.02.021.

85. Bourguignon LYW, Giland E, Peyrollier K, Brightman A, Swanson RA. Hyaluronan - CD44 interaction stimulates Rac1 signaling and PKNγ kinase activation leading to cytoskeleton function and cell migration in astrocytes. J Neurochem. 2007;101(4): 1002-1017. doi: 10.1111/j.1471-4159.2007.04485.x.

86. Thusberg J, Vihinen M. The structural basis of hyper IgM deficiency -

CD40L mutations. Protein Eng Des Sel. 2007;20(3): 133-41. doi: 10.1093/protein/gzm004

87. Coyne VE. The importance of ATP in the immune system of molluscs. ISJ. 2011;8: 48-55.

88. Anick DJ, Ives JA. The silica hypothesis for homeopathy: physical chemistry. Homeopathy. 2007;96: 189-195.007. doi: 10.1016/j.homp.2007.03.005.

89. Gonzalez-Muñoz MJ, Meseguer I, Sanchez-Reus MI, Schultz A, Olivero R, Benedí J, et al. Beer consumption reduces cerebral oxidation caused by aluminum toxicity by normalizing gene expression of tumor necrotic factor alpha and several antioxidant enzymes. Food Chem Toxicol. 2008;46: 1111-1118. doi: 10.1016/j.fct.2007.11.006.

90. Mazón-Suástegui JM, García-Bernal M, Abasolo-Pacheco F, Avilés-Quevedo A, Campa-Córdova AI, Rodríguez-Jaramillo C. Homeopathy for shrimp aquaculture: increased survival and supe-roxide dismutase activity in juvenile white shrimp *Litopenaeus vannamei* during a bacterial pathogen-challenge. Homeopathy. 2016;105: 33. doi: 10.1016/j.homp.2015.12.057.

91. Giovannini C, Baglioni M, Baron M, Cescon M, Bolondi L, Gramantieri L. Vidatox 30 CH has tumor activating effect in hepatocellular carcinoma. Sci

Rep. 2017;7: 44685. doi: doi.org/10.1038/srep44685.

92. Sullivan LB, Chandel NS. Mitochondrial reactive oxygen species and cancer. Can Metab. 2014;2: 17. doi: 10.1186/2049-3002-2-17

93. Appel HM, Cocroft RB. Plants respond to leaf vibrations caused by insect herbivore chewing. Oecologia. 2014;175(4): 257–1266. doi: 10.1007/s00442-014-2995-6.

94. Ghosh R, Mishra RC, Choi B, Kwon YS, Bae DW, Park CH, et al. Exposure to Sound Vibrations Lead to Transcriptomic, Proteomic and Hormonal Changes in *Arabidopsis*. Sci Rep. 2016;6: 33370. doi: 10.1038/srep33370.

Supporting information captions

S1 Fig. Expression patterns for cluster with functional enrichment with Gene Ontology (GO) or Kyoto Encyclopaedia of Genes and Genomes (KEGG) data base. Biological process or metabolic pathways with more than five transcripts are shown in graph with grey lines with the average datum behaviour represented by a red line.

S2 Fig. Biological process enrichment of Gene Ontology (GO) categories based on the differentially expressed genes (DEG) in each analysed contrast in mantle and gill tissue. The colour key indicates from beige to

brown the increasing percentages of genes representing each up-regulated category. The colour key indicates from beige to orange the increasing percentages of genes representing each down-regulated category. Blast2go specific filter was applied to the enrichment analysis to eliminate general categories. Contrasting conditions referred to all treatments evaluated vs control.

S3 Fig. Top 25 metabolic pathways enriched (Kyoto Encyclopaedia of Genes and Genomes) categories based on the differentially expressed genes (DEG) in each analysed contrast in mantle and gill tissue. The colour key indicates from beige to brown the increasing percentages of genes representing each up-regulated category. The colour key indicates from beige to orange the increasing percentages of genes representing each down-regulated category. Blast2go specific filter was applied to the enrichment analysis to eliminate general categories. Contrasting conditions referred to all treatments evaluated vs control.

S1 Table. Primers used to validate the transcriptome differentially expressed genes (DEG) analysis.

S2 Table. Total differentially expressed genes (DEG) in mantle and gill from scallops treated with highly-diluted immunomodulatory compounds (HDIC).

S3 Table. Differentially expressed genes (DEG) in mantle selected from

enriched biological process and metabolic pathways related to non-self-recognition, internalisation and immune response.

S4 Table. Differentially expressed genes (DEG) in gill selected from enriched biological process and metabolic pathways related to non-self-recognition, internalisation and immune response.
