# Supplementary material for: Transcriptome analysis of Catarina scallop (Argopecten ventricosus) juveniles treated with highly-diluted immunomodulatory compounds reveals activation of non-self-recognition system
Source: PLoS One. 2020 May 14;15(5):e0233064. doi: 10.1371/journal.pone.0233064 (PMC7224555; doi:10.1371/journal.pone.0233064)
Supplement: Attachment — Submitted filename: Dear editor.pdf [file pone.0233064.s009.pdf]

Dear editor,

I do hope that everything is OK. In respect of the novelty of the research paper that I had received for reviewing, I want to inform you that the manuscript (PONE-D-20-00449) entitled "**Transcriptome analysis of Catarina scallop (*Argopecten ventricosus*) juveniles treated with highly-diluted immunomodulatory compounds reveals activation of non-self-recognition system**" could be suitable for the processes of publication only after **Minor revision**.

This article will add a value in the literatures, however, I have only three comments in the materials and methods section.
